# Helminths of the marsh frog (*Pelophylax ridibundus*) from three localities in the city of Kyiv, Ukraine

**DOI:** 10.1017/S0031182026102169

**Published:** 2026-06

**Authors:** Ivanna Dmytriieva, Roman Svitin, Yuriy Kuzmin, Oleksiy Marushchak, Yaroslav Syrota, Tetiana Kuzmina

**Affiliations:** 1 I. I. Schmalhausen Institute of Zoology, National Academy of Sciences of Ukraine, Kyiv, Ukraine; 2 North-West University - Potchefstroom Campus, Potchefstroom, South Africa; 3 Taras Shevchenko National University of Kyiv, Kyiv, Ukraine; 4 Institute of Parasitology, Slovak Academy of Sciences, Košice, Slovakia

**Keywords:** abundance, diversity indices, helminth community, species richness, urbanization

## Abstract

The study aimed to characterize the species composition and compare helminth populations and communities in samples of helminths collected from marsh frogs in 3 localities in the city of Kyiv. Twenty helminth species were recorded in total, of which 8 were found in all 3 localities: the nematodes *Cosmocerca ornata* and *Icosiella neglecta*, and the trematodes *Diplodiscus subclavatus, Haematoloechus asper, Opisthioglyphe ranae, Pleurogenes claviger, Pleurogenoides medians* and *Prosotocus confusus*. In helminth infracommunities, species richness and abundance were the highest in frogs from Olzhyn Island (median 7 and 98, respectively). Helminth species richness was the lowest in the infracommunities from Lake Tiahle (median 4), while the abundance was the lowest in the infracommunities from Lake Telbin (median 51.5). From 3 to 4 species predominated in separate helminth component communities based on their high prevalence and abundance in the studied samples: *D. subclavatus, I. neglecta, P. medians* and *P. confusus* in Lake Telbin; *Codonocephalus urniger, I. neglecta, O. ranae* and *P. confusus* in Lake Tiahle; *C. urniger, O. ranae* and *Tylodelphys excavata* in Olzhyn Island. According to our observations, intense urbanization of Lake Telbin affects rather the abundance than the species richness in helminth communities of the marsh frog. On the other hand, the relatively low abundance of helminths in the infrapopulations from Lake Telbin resulted in comparatively higher diversity indices for the helminth component community in this locality. Moderate urbanization, on the contrary, results in the strong predominance of some helminth species and low values of the diversity indices.

## Graphical abstract

**Figure:**
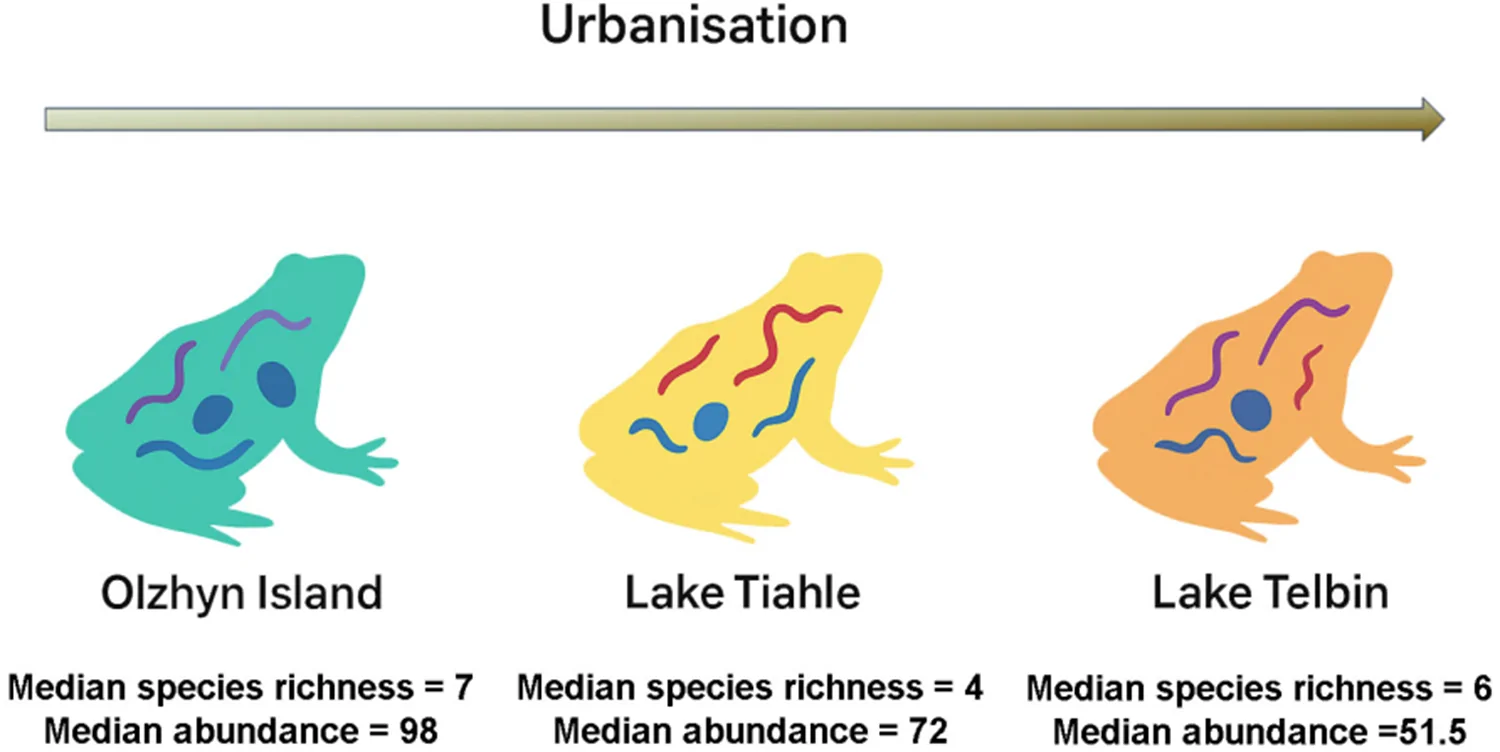

## Introduction

Parasites may serve as indicators of environmental conditions in ecosystems; moreover, it has been suggested that healthy ecosystems are rich in parasites (Marcogliese, 2005; Hudson et al., 2006). Therefore, studies of parasite communities, even within a particular host population or group of populations of ecologically close hosts, may provide valuable insights into the state and health of a particular ecosystem. Parasites with complex life cycles are especially useful as indicators of ecosystem health, as they require the presence of a variety of different organisms that serve as intermediate, paratenic and definitive hosts, as well as the trophic interactions necessary for transmission (Marcogliese, 2023). Additionally, environmental conditions directly influence the free-living stages of parasites, including those with monoxenous life cycles.

Habitat modification, as one of the threats to biodiversity worldwide, inevitably affects parasite populations and communities. Urbanization, for instance, may reduce the diversity and number of intermediate invertebrate hosts for heteroxenous parasites of vertebrates (King et al., 2007), thereby reducing the species richness in parasite communities. It was suggested that, in general, effects of habitat modifications on parasite communities are ‘negative, but difficult to predict’ (Marcogliese, 2023).

Helminth populations and communities in amphibians are a promising subject for ecological studies. Amphibians are generalist predators, on the one hand, and prey to larger predators (mainly birds and reptiles), on the other hand; due to this, they are intermediate, paratenic and definitive hosts for a variety of parasites in aquatic and terrestrial food webs (Koprivnikar et al., 2012). Aquatic and semi-aquatic species of amphibians usually harbour a diverse helminth fauna due to the presence of heteroxenous helminths (mostly trematodes) with aquatic intermediate hosts (molluscs). This fact makes them a potential model group for investigating parasite ecology and the effects of habitat changes on parasite communities. For example, parasite species richness, both in infracommunities and in component communities, was shown to be lower in agricultural and urban areas than in natural ecosystems (King et al., 2007, 2010).

The marsh frog *Pelophylax ridibundus* (Pallas, 1771) is a semi-aquatic amphibian species widely distributed in the Western Palaearctic (Frost, 2025). It usually inhabits large water bodies (rivers, lakes, ponds and dams) in open areas (Pysanets, 2014) and is rather tolerant of habitat changes caused by human activities (Pysanets, 2007). Helminths of the marsh frog, often along with other anuran hosts, have been studied in various regions of Ukraine by Mazurmovich (1951), Volgar-Pastukhova (1959), Maguza (1972), Rezvantseva et al. (2010), Kuzmin et al. (2020) and Greben et al. (2025), mainly in natural or moderately transformed habitats. Similar studies have been conducted in Poland (Popiołek et al., 2011), Hungary (Herczeg et al., 2016) and Belarus (Shimalov, 2009). In Russia, several studies on the helminths of the marsh frog have been performed, including in urbanized areas (e.g. Chihlaev et al., 2009; Vershinin et al., 2017).

We investigated helminth infections in the marsh frogs from 3 urbanized localities in the city of Kyiv: Lake Telbin (within the city), Lake Tiahle and the bank of the Dnipro River near Olzhyn Island; the latter 2 localities are situated at the southern margin of the city. The aim of the study was to identify the species composition of the 3 helminth samples studied and to characterize and compare helminth populations and communities in marsh frogs from the 3 localities.

## Materials and methods

### Study areas

**Figure 1.**
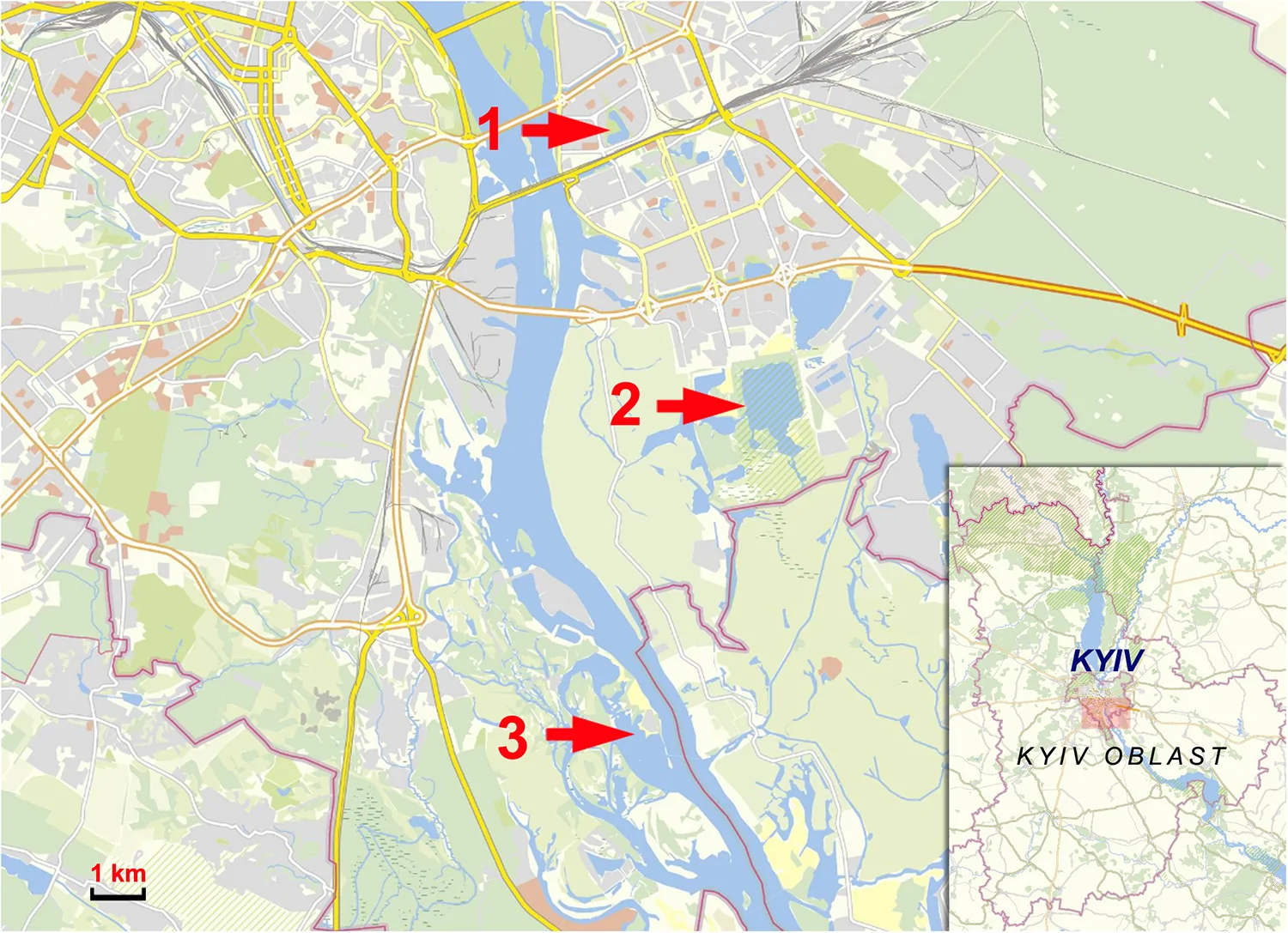
Map of collection sites: 1 – Lake Telbin, 2 – Lake Tiahle, 3 – Olzhyn Island. Figure 1 long description.

Lakes Telbin and Tiahle are situated on the eastern bank of the Dnipro River (Figure 1). Both lakes were affected by the construction of residential areas in the south-eastern part of the city (Berezniaky, Rusanivka and Pozniaky districts) in 1960–1980. During construction, the area of Lake Telbin was reduced; it was separated from the Dnipro by a 2–3 m high elevation of the bank and surrounded by streets, roads and multi-storey residential buildings. Despite the extensive human activities around the lake (picnicking, fishing and bathing at 2 beaches on the south-western bank), it still harbours a population of marsh frogs, as well as European pond turtles, *Emys orbicularis* (Linnaeus, 1758) and several species of birds, mainly waterfowl.

Lake Tiahle was used as a sand pit during the construction of residential districts from 1980 to 1995 and was consequently enlarged and deepened (Vyshnevskyi, 2021). It borders Lakes Vyrlytsia and Nebrezh; the whole lake system harbours significant populations of marsh frogs and waterfowl. Human activities on the lake’s banks are restricted to camping, picnicking and recreational fishing.

The third collection site is located on the bank of the river inlet to the north of Olzhyn Island on the western bank of the Dnipro River. Human activities have not severely modified the area around. Populations of the marsh frogs, the grass snakes *Natrix natrix* (Linnaeus, 1758) and various birds suffer primarily from disturbance caused by people camping, picnicking and fishing.

### Host and parasite collection and identification

**Figure 2.**
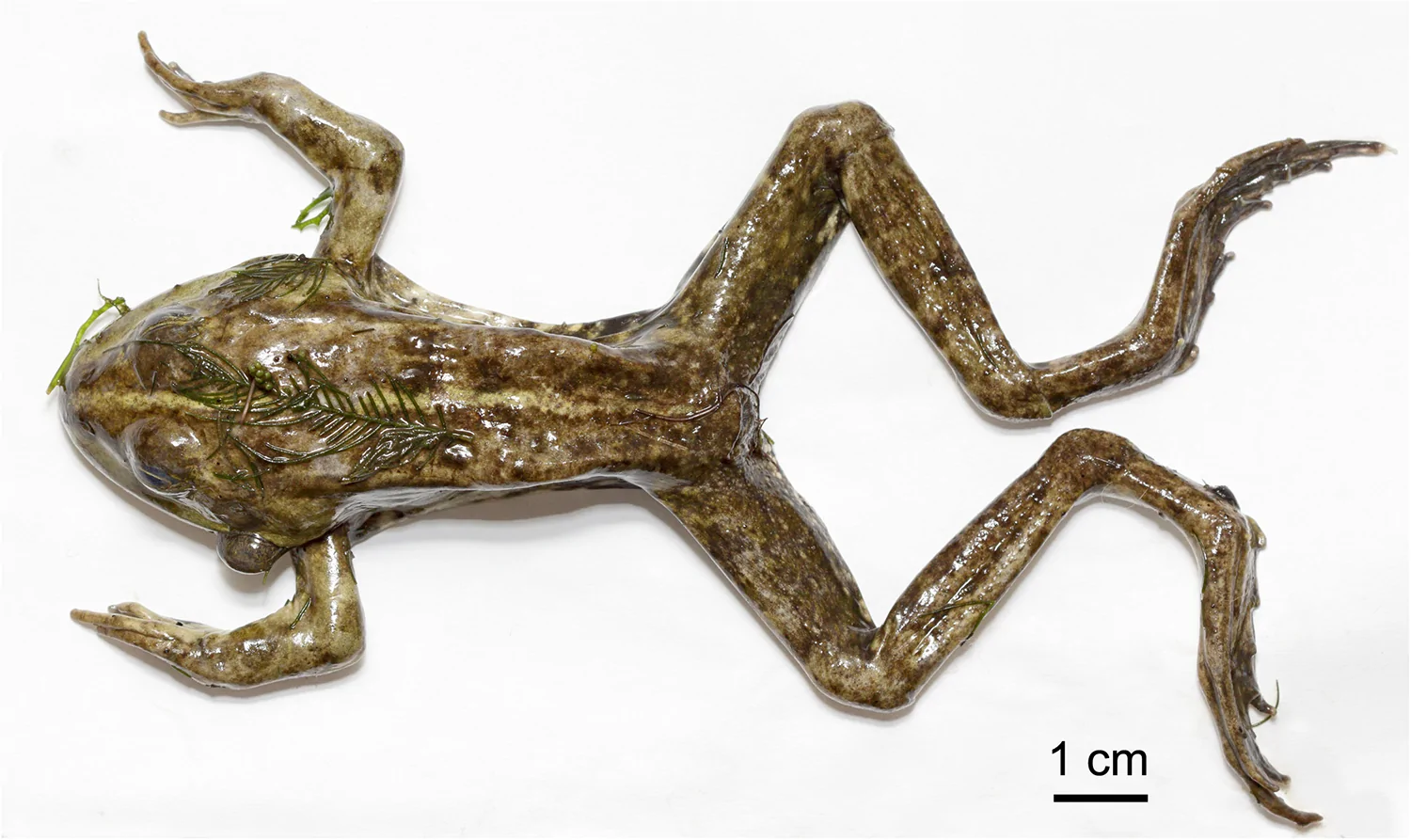
Collected frog from Lake Telbin with signs of dystrophy and exhaustion.

Frogs were collected manually or with a net, either during the daytime or at night with a torch, and thereafter transferred to the laboratory. All collected frog individuals were identified as *P. ridibundus* based on morphological characters (Nekrasova, 2002; Nekrasova et al., 2004; Pysanets, 2007). Sites of collection and parameters of each frog sample are given in Table 1. It is noteworthy that some collected frogs from Lake Telbin showed the signs of exhaustion and dystrophy (Figure 2); moreover, frogs from this locality often had inedible items in their stomachs: cherry pits, feathers, pieces of plastic, etc., which evidenced the restriction of food available for frogs and their consequent starvation. In the laboratory, frogs were measured, euthanized by lidocaine injection into the brain stem, autopsied and examined under a stereomicroscope.

**Table 1.** Collection localities, dates and parameters of collected samples of the marsh frogs (*Pelophylax ridibundus*) Table 1 long description.

Collection locality (coordinates)	Month and year(s) of collection	Number of host specimens (females/males)	Snout–vent length of hosts, mm*
Lake Telbin (50.425 N, 30.610 E)	September 2018, 2019	24 (8/16)	82 ± 10 (58–102)
Lake Tiahle (50.380 N, 30.640 E)	June–September 2020	33 (16/17)	74 ± 14 (43–99)
Olzhyn Island (50.328 N, 30.611 E)	July 2021	20 (10/10)	68 ± 13 (47–92)

* Presented as mean value ± SD (minimum–maximum).

Collected nematodes and trematodes were washed in saline and fixed with hot 70% ethanol. Acanthocephalans were kept in distilled water for 2–24 h till the complete evagination of the proboscis and relaxation of the trunk, and then fixed with 70% ethanol. Collected specimens were cleared in lactophenol for 0.5–2 h and examined under light microscopes AmScope T690B and Zeiss AxioImager M1, equipped with a digital imaging system and differential interference contrast. Photomicrographs were produced using the digital imaging system of the Zeiss AxioImager M1 microscope.

### Characteristics of the helminth populations and communities

For each helminth species in each sample, the infection prevalence (P), intensity (I) and mean abundance (MA) were calculated following the definitions of Bush et al. (1997). Additionally, the relative abundance (RA) of each species was calculated as the percentage of its specimens in the whole sample of helminths. The dispersion index (DI) was calculated as the variance-to-mean ratio following the recommendations of Rózsa et al. (2000). We calculated 95% confidence intervals for infection prevalence and MA using the Quantitative Parasitology 3.0 software (Rózsa et al., 2000). The same program was used to compare infection prevalence (using the unconditional exact test, UET) and MA (using the bootstrap-*t* test, BTT) across separate species and samples. A species was classified as statistically uncertain in each sample if the minimum value of the confidence interval of its infection prevalence was lower than the lowest possible value of infection prevalence in the sample, and/or if the minimum value of the confidence interval of its MA equalled 0.

In helminth infracommunities, species richness and helminth abundance were identified, and mean and median values were calculated for each sample. Median values of species richness and abundance in the infracommunities from separate samples were compared; the significance of the differences was identified using the Mann–Whitney *U*-test in PAST v.3.10 (Hammer, 1999–2015). The correlation between host body size and helminth infracommunity descriptors was identified using the Spearman’s correlation coefficient calculated in PAST v.3.10. Additionally, the relationship between host body size and helminth infracommunity descriptors was analysed using generalized linear models (GLMs) in R v.4.4.2 (R Core Team, 2024). A Poisson GLM was fitted for helminth species richness with the stats package, while a negative binomial GLM was used for the abundance with the MASS package (Venables and Ripley, 2002). Model diagnostics were performed by checking simulated residuals using the performance package (Lüdecke et al., 2021). The similarity between helminth communities from each locality was estimated using the Bray–Curtis index in PRIMER 6 (Clarke and Gorley, 2006). Prior to the similarity calculation, the sampled data were modified using log(*x* + 1) transformation, and samples were standardized by total. The same program was used to visualize similarities among infracommunities using non-metric multidimensional scaling (nMDS) and to estimate similarities and differences among infracommunities from separate samples using SIMPER and ANOSIM. The diversity indices and the estimated species richness (based on *Chao1, jackknife1* and *bootstrap* methods) for the helminth component communities in each sample were also calculated in PRIMER 6 software.

## Results

### Helminth species and populations

Twenty helminth species were collected from marsh frogs in 3 localities, including 1 species of acanthocephalans, 5 species of nematodes and 14 species of trematodes (Table 2). Four helminth species were found as juvenile stages (third-stage larvae of *Spiroxys contortus* (Rudolphi, 1819), metacercariae of *Plesioastiotrema monticelli* (Stossich, 1904), *Codonocephalus urniger* (Rudolphi, 1819) and *Tylodelphys excavata* (Rudolphi, 1803)); the remaining 16 species were represented by adult stages.

**Table 2.** Infection parameters of helminth species found in *Pelophylax ridibundus* from 3 localities Table 2 long description.

		Lake Telbin (*n* = 24)			Lake Tiahle (*n* = 33)			Olzhyn Island (*n* = 20)		
	Species	P, %	MA	I	P, %	MA	I	P, %	MA	I
Acanthocephala: Palaeacanthocephala										
1.	*Acanthocephalus ranae* (Schrank, 1788)	0	0	0	12.1 (4.2–28.4)	0.12 (0.03–0.24)	1 (1–1) 1	60 (37.2–79.1	1.15 (0.6–1.75)	1.9 (1–4) 1
Nematoda: Chromadorea										
2.	*Cosmocerca ornata* (Dujardin, 1845)	58.3 (37.2–76.6)	1.54 (0.79–2.63)	2.6 (1–7) 1	9.1 (2.5–23.9)	0.09 (0–0.18)	1 (1–1) 1	60 (37.2–79.1)	1.1 (0.65–1.9)	1.8 (1–6) 1.5
3.	*Icosiella neglecta* (Diesing, 1851)	87.5 (69–96.5)	6.25 (4.42–8.63)	7.1 (1–20) 6	84.8 (68.4–93.8)	6.21 (4.58–8.55)	7.3 (1–24) 6	20 (7.1–42.3)	1.05 (0.1–4.35)	5.25 (1–17) 1.5
4.	*Neyraplectana schneideri* (Travassos, 1931)	4.2 (0.2–20.4)	0.04 (0–0.13)	1 (1–1) 1	6.1 (1.1–19.4)	0.06 (0–0.15)	1 (1–1) 1	0	0	0
5.	*Spiroxys contortus* (Rudolphi, 1819) (L3)	4.2 (0.2–20.4)	0.04 (0–0.13)	1 (1–1) 1	0	0	0	0	0	0
6.	*Strongyloides spiralis* (Grabda-Kazubska, 1978)	4.2 (0.2–20.4)	0.42 (0–1.25)	10 (10–10) 10	0	0	0	5 (0.3–24.4)	0.05 (0–0.15)	1 (1–1) 1
Platyhelminthes: Trematoda										
7.	*Cephalogonimus retusus* (Dujardin, 1845)	12.5 (3.5–31)	0.17 (0–0.42)	1.3 (1–2) 1	0	0	0	30 (14–52.5)	1.4 (0.45–3.85)	4.7 (1–14) 3.5
8.	*Codonocephalus urniger* (Rudolphi, 1819) (mtc)	0	0	0	72.7 (54.6–85.3)	26.88 (17.27–44.33)	37 (1–169) 23.5	85 (62.8–95.8)	15.55 (9.45–25.75)	18.3 (1–67) 12
9.	*Diplodiscus subclavatus* (Pallas, 1760)	87.5 (69–96.5)	9 (6.33–12.54)	10.3 (1–31) 9	48.5 (31.6–65.3)	1.88 (0.97–3.76)	3.9 (1–18) 2	60 (37.2–79.1)	1.3 (0.7–3.1)	2.2 (1–5) 1.5
10.	*Gorgodera cygnoides* (Zeder, 1800)	0	0	0	3 (0.2–16.1)	0.06 (0–0.18)	2 (2–2) 2	0	0	0
11.	*G. pagenstecheri* (Sinitzin, 1905)	20.8 (8.6–41.5)	0.25 (0.04–0.46)	1.2 (1–2) 1	0	0	0	0	0	0
12.	*Haematoloechus asper* (Looss, 1899)	4.2 (0.2–20.4)	0.04 (0–13)	1 (1–1) 1	15.1 (6.2–31.6)	0.33 (0.09–0.67)	2.2 (1–3) 3	30 (14–52.5)	1.8 (0.5–4.6)	6 (1–16) 4
13.	*H. variegatus* (Rudolphi, 1819)	0	0	0	3 (0.2–16.1)	0.03 (0–0.09)	1 (1–1) 1	5 (0.3–24.4)	0.05 (0–0.15)	1 (1–1) 1
14.	*Opisthioglyphe ranae* (Froelich, 1791)	50 (31–69)	2.21 (1.08–3.92)	4.4 (1–11) 3.5	90.9 (76.1–97.5)	32.61 (21.12–52.52)	35.9 (4–209) 16	100 (83.3–100)	172.7 (50.1–627.25)	172.7 (2–2326) 37.5
15.	*Plesioastiotrema monticelli* (Stossich, 1904) (mtc)	0	0	0	3 (0.2–16.1)	0.03 (0–0.09)	1 (1–1) 1	0	0	0
16.	*Pleurogenes claviger* (Rudolphi, 1819)	66.7 (45.8–83.1)	1.62 (1.04–2.21)	2.4 (1–4) 2.5	24.2 (11.7–42.3)	0.58 (0.24–1.21)	2.4 (1–6) 2	30 (14–52.5)	1.15 (0.35–3.35)	3.8 (1–13) 2
17.	*Pleurogenoides medians* (Olsson, 1876)	70.8 (50–86.1)	4.83 (2.96–8.04)	6.8 (1–26) 5	21.2 (10.1–37.8)	1.27 (0.21–3.58)	6 (1–18) 1	50 (29.3–70.7)	1.15 (0.55–2.65)	2.3 (1–9) 1.5
18.	*Prosotocus confusus* (Looss, 1894)	79.2 (58.5–91.4)	29.58 (19.58–40.79)	37.4 (2–88) 30	42.4 (26.8–59.9)	9.82 (4.85–18.18)	23.1 (2–72) 11.5	65 (42.4–83.3)	9.25 (4.85–15.95)	14.2 (1–42) 9
19.	*Skrjabinoeces similis* (Looss, 1899)	41.7 (23.4–62.7)	1.54 (0.71–3.33)	3.7 (1–13) 2	0	0	0	0	0	0
20.	*Tylodelphys excavata* (Rudolphi, 1803) (mtc)	0	0	0	3 (0.2–16.1)	0.27 (0–0.82)	9 (9–9) 9	90 (68–90.2)	52.7 (23.35–137.05)	58.6 (1–435) 10.5

P, Prevalence of infection; MA, mean abundance; I, intensity of infection; mtc, metacercaria; L3, third-stage larva; 95% confidence intervals for P and MA are given in parentheses; infection intensity is presented as mean value followed by range in parentheses and median value.

A similar number of helminth species was recorded in frog samples from the 3 localities: 14 in Lake Telbin, 15 in Lake Tiahle and 14 in Olzhyn Island. Eight species were found in all 3 localities: the nematodes *Cosmocerca ornata* (Dujardin, 1845) and *Icosiella neglecta* (Diesing, 1851), and the trematodes *Diplodiscus subclavatus* (Pallas, 1760), *Haematoloechus asper* Looss, 1899, *Opisthioglyphe ranae* (Froelich, 1791), *Pleurogenes claviger* (Rudolphi, 1819), *Pleurogenoides medians* (Olsson, 1876) and *Prosotocus confusus* (Looss, 1894) (Table 2). The trematodes *Gorgodera pagenstecheri* Sinitzin, 1905 and *Skrjabinoeces similis* (Looss, 1899), and the nematode *S. contortus* were found only in the sample from Lake Telbin. The trematodes *P. monticelli* and *Gorgodera cygnoides* (Zeder, 1800) were present only in the sample from Lake Tiahle. No species unique to the hosts from Olzhyn Island were recorded.

Based on species composition, the highest similarity was observed between the hosts from Lake Tiahle and Olzhyn Island, with 12 common species (Sorensen index 0.83). The samples from Lake Telbin and Olzhyn Island shared 10 species (Sorensen index 0.71). The lowest similarity was observed between the samples from Lake Telbin and Lake Tiahle: 9 common species (Sorensen index 0.62).

Presented below is brief information about each species of helminths, along with a comparison of the parameters of helminth component populations in 3 localities.

**Phylum Acanthocephala**

**Class Palaeacanthocephala**

*Acanthocephalus ranae* (Schrank, 1788)

The species was found in the small intestine of 4 frogs from Lake Tiahle (4 specimens in total) and 12 frogs from Olzhyn Island (23 specimens in total); all infection parameters were higher at the latter locality (Table 2). The differences between the 2 samples in infection prevalence and MA were significant (UET *p* < 0.01, BTT *p* = 0.02, correspondingly). In the sample from Lake Tiahle, *A. ranae* did not demonstrate aggregated distribution (DI = 0.91); each infected host individual harboured a single specimen of the parasite.

**Phylum Nematoda**

**Class Chromadorea**

*Cosmocerca ornata* (Dujardin, 1845)

The species was found in the rectum and large intestine of frogs at all 3 localities, with a total of 37 specimens in the sample from Lake Telbin, 3 specimens in the sample from Lake Tiahle and 22 specimens in the sample from Olzhyn Island. Infection prevalence and abundance of *C. ornata* were higher and similar in Lake Telbin and Olzhyn Island (UET, *p* = 1 for infection prevalence; BTT, *p* = 0.44 for MA) and significantly lower in Lake Tiahle (Table 2). Besides, the distribution of *C. ornata* in frogs from Lake Tiahle was not aggregated in the studied sample (DI = 0.94), since each of the infected hosts harboured 1 specimen of the parasite.

**Figure 3.**
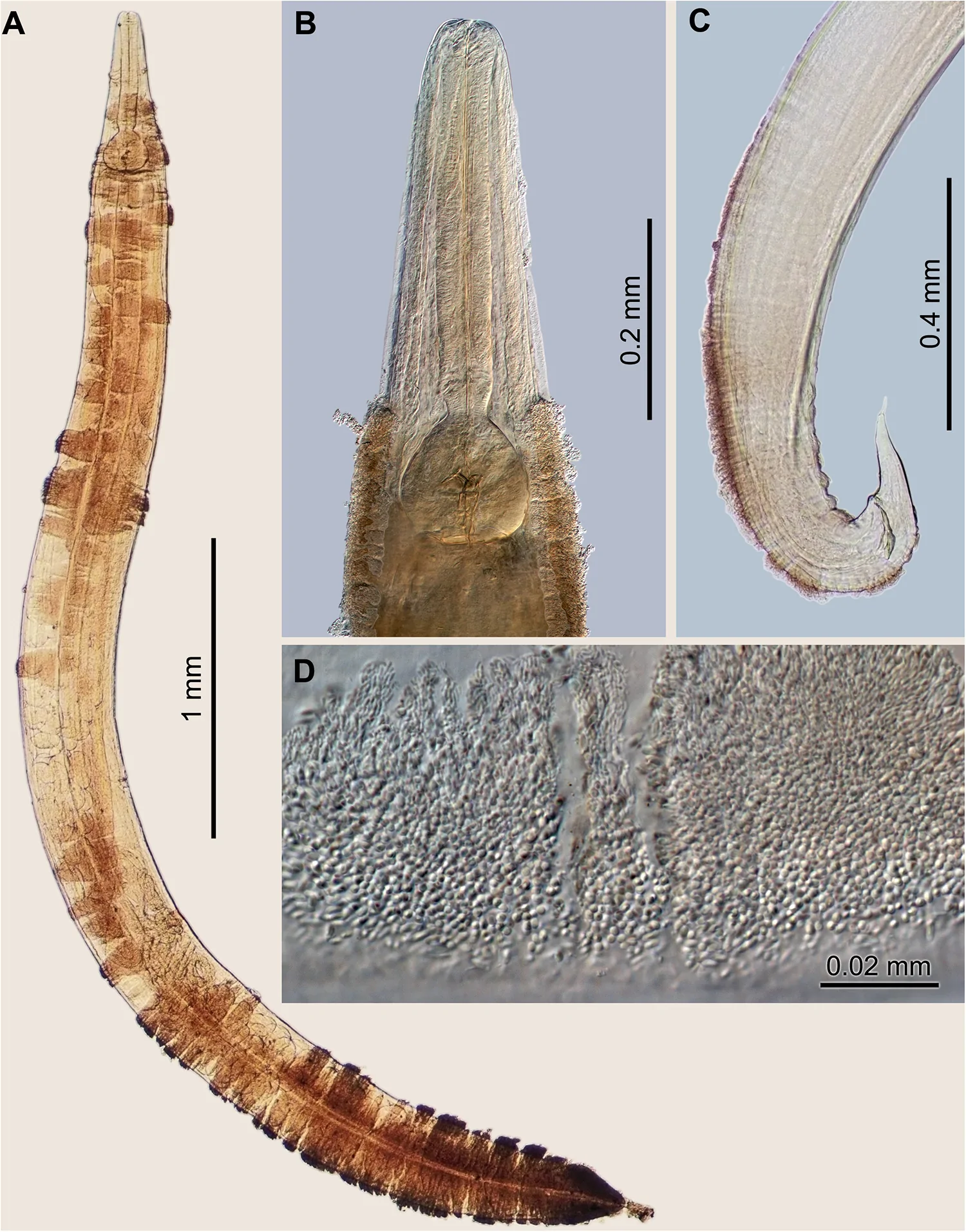
*Cosmocerca ornata* from Lake Telbin: A – female, general view, B – female, anterior part of body, C – male, posterior part of body, D – surface of moss-like patches on cuticle. Figure 3 long description.

In the sample from Lake Telbin, the body cuticle of all specimens of *C. ornata* was damaged, presumably because of bacterial or fungal infection. The cuticle appeared dark with moss-like patches scattered across the body’s surface, except for its anterior part (Figure 3).

*Icosiella neglecta* (Diesing, 1851)

These muscle-dwelling nematodes were found in the limb and throat muscles, rarely in the body cavity of frogs, in all 3 localities. In total, 150 specimens were collected in the sample from Lake Telbin, 205 specimens were from Lake Tiahle and 21 specimens were from Olzhyn Island. Infection prevalence and MA of *I. neglecta* were almost identical in frog populations from Lake Telbin and Lake Tiahle (UET *p* = 1, BTT *p* = 0.98, correspondingly). Both parameters were significantly lower in the population from Olzhyn Island compared to 2 former localities (Table 2), as indicated by statistical tests: UET and BTT *p* < 0.01 in both pairwise comparisons. In the sample from Lake Telbin, several dead specimens of *I. neglecta* were observed in each of the most heavily infected frogs.

*Neyraplectana schneideri* (Travassos, 1931)

One male of the species was found in the rectum of 1 frog from Lake Telbyn; 2 frogs from Lake Tiahle were infected with 1 male of *N. schneideri* each. Co-infection with *C. ornata* (male) was observed in a frog from Lake Telbin.

*Spiroxys contortus* (Rudolphi, 1819)

One encysted third-stage larva of *S. contortus* was found in the intestine wall of a frog from Lake Telbin.

*Strongyloides spiralis* (Grabda-Kazubska, 1978)

Ten specimens of *S. spiralis* were found embedded in the rectal mucosa of 1 frog from Lake Telbin; 1 specimen was found in a frog from Olzhyn Island. Morphologically, all specimens appeared to correspond to the description of *S. spiralis* from *Pelophylax* spp. in Poland (Grabda-Kazubska, 1978).

**Phylum Platyhelminthes**

**Class Trematoda, Subclass Digenea**

*Cephalogonimus retusus* (Dujardin, 1845)

Four specimens of *C. retusus* were collected from the small intestine of 3 frogs from Lake Telbin. In the sample from Olzhyn Island, 6 frogs (30%) were infected with a total of 28 specimens of the species. The difference in infection prevalence and MA in the 2 samples was not significant: UET *p* = 0.18, BTT *p* = 0.23, correspondingly. On the other hand, the infection intensity was higher in the sample from Olzhyn Island, reaching 14 specimens in 1 frog (Table 2).

*Codonocephalus urniger* (Rudolphi, 1819)

Numerous metacercariae of *C. urniger* were observed in frogs from Lake Tiahle (887 specimens in total) and Olzhyn Island (311 specimens in total); they were absent in the sample from Lake Telbin. Cysts with metacercariae were found throughout the body cavity, attached to large nerves and the outer surface of internal organs. In the most heavily infected frogs, cysts were also found in the limb and body muscles, in the liver and under the skin. The infection prevalence was slightly higher in the sample from Olzhyn Island, while the MA was higher in the sample from Lake Tiahle (Table 2). The differences, however, were not significant: UET *p* = 0.38, BTT *p* = 0.16, correspondingly.

*Diplodiscus subclavatus* (Pallas, 1760)

The species was found in the posterior part of the intestine and in the rectum of frogs in all 3 localities. It was more numerous in the sample from Lake Telbin (216 specimens in total) than in those from Lake Tiahle (62 specimens in total) and Olzhyn Island (26 specimens in total). The prevalence of infection and MA were the highest in Lake Telbin, while the lowest infection prevalence was in Lake Tiahle, and the lowest MA was in the sample from Olzhyn Island (Table 2). The difference in infection prevalence between the sample from Lake Telbin and the 2 other samples was significant (UET *p* < 0.01 and UET *p* = 0.04, correspondingly). Similarly, a significant difference was observed between the sample from Lake Telbin and the other 2 samples in the MA of *D. subclavatus* (BTT *p* < 0.01). Differences in infection prevalence and MA between the sample from Lake Tiahle and the sample from Olzhyn Island were not significant (UET *p* = 0.44, BTT *p* = 0.45, correspondingly).

*Gorgodera cygnoides* (Zeder, 1800)

Two specimens of *G. cygnoides* were found in the bladder of 1 frog from Lake Tiahle.

*Gorgodera pagenstecheri* Sinitzin, 1905

Six specimens of *G. pagenstecheri* were found in the bladder of 5 frogs from Lake Telbin.

*Haematoloechus asper* Looss, 1899

The species was found in the lungs of frogs from Lake Telbin (1 specimen), Lake Tiahle (11 specimens in total) and Olzhyn Island (36 specimens in total). The infection prevalence and MA were the highest in the sample from Olzhyn Island and the lowest in the sample from Lake Telbin (Table 2). The difference in the infection prevalence between the samples from Lake Telbin and Olzhyn Island was significant (UET *p* = 0.03). Co-occurrence of *H. asper* with other lung-dwelling trematodes was not observed in any of the samples.

*Haematoloechus variegatus* (Rudolphi, 1819)

The species was found in the lungs of 1 frog from Lake Tiahle (1 specimen) and 1 frog from Olzhyn Island (1 specimen).

*Opisthioglyphe ranae* (Frohlich, 1791)

The species was found in the intestine of frogs from Lake Telbin (53 specimens in total), Lake Tiahle (1076 specimens in total) and Olzhyn Island (3454 specimens in total). High infection intensity in the latter 2 localities (up to 2326 specimens in 1 frog) was primarily due to the large numbers of young (immature) specimens of the trematode in frog intestines. The infection prevalence of 50% in the sample from Lake Telbin was the lowest among the 3 samples (Table 2), while in the sample from Olzhyn Island, all frogs were infected. The differences in infection prevalence and MA between the sample from Lake Telbin and 2 other localities were significant (UET and BTT *p* < 0.01).

*Plesioastiotrema monticelli* (Stossich, 1904)

One metacercaria of *P. monticelli* was found encysted in the body cavity of a frog from Lake Tiahle.

*Pleurogenes claviger* (Rudolphi, 1819)

The specimens of *P. claviger* collected from the frogs’ small intestine were more numerous in the sample from Lake Telbin (39 specimens in total) than from Lake Tiahle (19 specimens) and Olzhyn Island (23 specimens). The infection prevalence of *P. claviger* was significantly higher in the sample from Lake Telbin than in the other 2 samples: UET *p* < 0.05 in both pairwise comparisons. In the samples from Lake Tiahle and Olzhyn Island, the infection prevalence was statistically equal (UET *p* = 1). The difference in MA of *P. claviger* between the samples from Lake Telbin and Lake Tiahle was significant (BTT *p* < 0.01); it was not significant in other pairwise comparisons. Interestingly, the infection intensity of *P. claviger* was relatively similar in all 3 samples, with a slight increase in the sample from Lake Telbin (Table 2).

*Pleurogenoides medians* (Olsson, 1876)

The species was found in the small intestine of frogs from all 3 localities. In total, 116 specimens were found in the sample from Lake Telbin, 42 specimens were from Lake Tiahle and 23 specimens were from Olzhyn Island. The infection prevalence was the highest in the sample from Lake Telbin and the lowest in the sample from Lake Tiahle (Table 2); the difference between the 2 samples was significant (UET *p* < 0.01). Significant difference in the *P. medians* infection prevalence was also observed between the samples from Lake Tiahle and Olzhyn Island (UET *p* = 0.03). MA of *P. medians* was the highest in the sample from Lake Telbin and significantly different from that in the other 2 samples (BTT *p* = 0.02). In the samples from Lake Tiahle and Olzhyn Island, the MA of *P. medians* was similar (BTT *p* = 0.48); however, the species reached a higher infection intensity in frogs from the former locality (Table 2).

*Prosotocus confusus* (Looss, 1894)

The species was found in the anterior part of the small intestine, and occasionally in the stomach of frogs from all 3 localities. In total, 710 specimens were present in the sample from Lake Telbin, 324 specimens were from Lake Tiahle, and 185 specimens were from Olzhyn Island. Significant difference in the *P. confusus* infection prevalence was found only between the samples from Lake Telbin and Lake Tiahle (UET *p* < 0.01). MA of the species was significantly higher in the sample from Lake Telbin than in the other 2 samples (BTT *p* < 0.01). In the samples from Lake Tiahle and Olzhyn Island, the MA of *P. confusus* was rather similar: 9.82 and 9.25, correspondingly; BTT *p* = 0.9. Infection intensity of *P. confusus* was also markedly higher in the sample from Lake Telbin; its median value was approximately 3 times higher than in 2 other localities (Table 2).

*Skrjabinoeces similis* (Looss, 1899)

In total, 37 specimens of *S. similis* were collected from the lungs of 10 frogs from Lake Telbin. The infection intensity reached a maximum of 13 specimens in 1 frog, and the MA was 1.54 (Table 2).

*Tylodelphys excavata* (Rudolphi, 1803)

Motile metacercariae of *T. excavata* were found in the spinal canal, rarely in the body cavity, stomach and intestine of frogs in the samples from Lake Tiahle (9 specimens in 1 infected frog) and Olzhyn Island (1054 specimens in total; 90% of frogs infected).

### Helminth infracommunities

All 77 examined frog individuals from 3 localities were found to harbour at least 1 helminth specimen. In the combined sample, the species richness in helminth infracommunities ranged from 1 to 11 (mean 5.5, median 6), and the helminth abundance ranged from 1 to 2871 (mean 120, median 71). Both parameters showed a positive correlation with frog size; Spearman’s correlation coefficients were 0.38 (*p* < 0.01) and 0.36 (*p* < 0.01), respectively. Besides, both richness (GLM: *β* = 0.012, *z* = 3.35, *p* < 0.001) and abundance (GLM: *β* = 0.042, *z* = 4.73, *p* < 0.001) showed a significant positive association with frog size.

Species richness and helminth abundance varied among samples from separate localities (Table 3). On average, the highest species richness (median 7) and abundance (median 98) were observed in the helminth infracommunities from Olzhyn Island. In the helminth infracommunities from Lake Telbin, species richness was similar to that in the sample from Olzhyn Island (median 6), whereas abundance was the lowest among the 3 localities (median 51.5). In the sample from Lake Tiahle, the species richness in helminth infracommunities was the lowest (median 4), while the abundance was intermediate between the other 2 samples (median 72). A significant difference in species richness among helminth infracommunities from Lake Tiahle compared to 2 other localities was confirmed by the Mann–Whitney *U*-test (*p* < 0.01). A significant difference in median abundance was observed only between the infracommunities from Lake Telbin and Olzhyn Island (Mann–Whitney *U*-test *p* < 0.01).

**Table 3.** Species richness and abundance in helminth infracommunities of marsh frogs from 3 localities Table 3 long description.

	Species richness			Abundance		
Locality	Mean	Range	Median	Mean	Range	Median
Lake Telbin	5.9	2–8	6	57.5	2–129	51.5
Lake Tiahle	4.4	1–9	4	80.2	1–246	72
Olzhyn Island	6.9	3–11	7	260.4	17–2871	98

**Figure 4.**
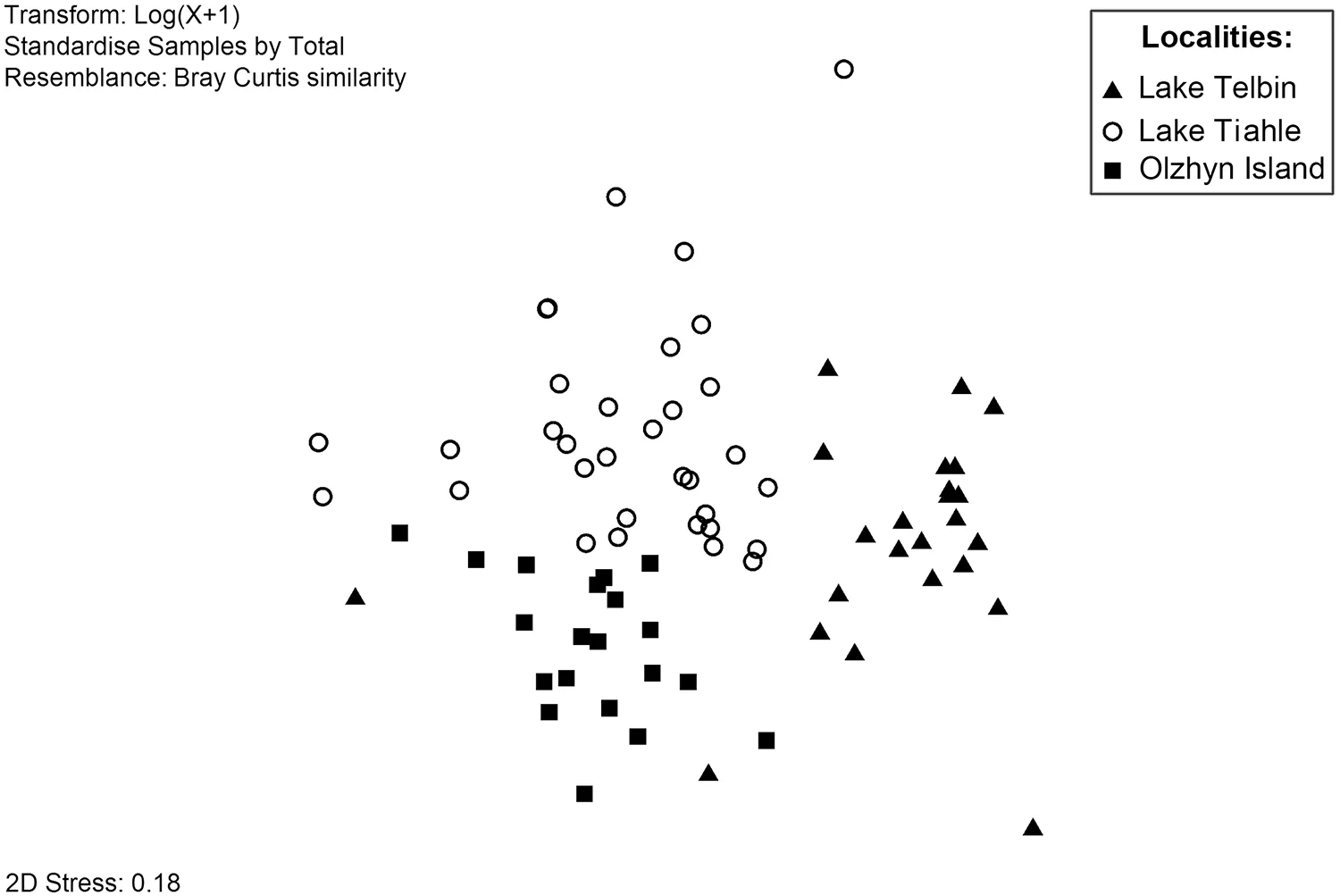
Two-dimensional view of nMDS distribution of helminth infracommunities from 3 localities based on Bray–Curtis similarity. Figure 4 long description.

In the combined sample, the similarity between individual helminth infracommunities ranged from 0 to 0.93, according to the Bray–Curtis similarity index. Visualization of the similarities using nMDS (Figure 4) demonstrated that the samples from separate localities formed distinct groups, though not markedly separated from one another. According to SIMPER analysis, the highest average ingroup similarity was between the individual infracommunities from Lake Telbin (40.4%), and the lowest one was in the sample from Lake Tiahle (31.3%); in the sample from Olzhyn Island, the ingroup similarity was 35.8%. According to the ANOSIM routine, the highest dissimilarity was observed between the infracommunities from Lake Telbin and Olzhyn Island (ANOSIM *R* = 0.79; *p* < 0.01).

SIMPER analysis identified *O. ranae, P. confusus, C. urniger* and *T. excavata* as the species with the highest contribution (>10%) to that dissimilarity. The lowest dissimilarity was observed between the infracommunities from Lake Tiahle and Olzhyn Island (ANOSIM *R* = 0.34; *p* < 0.01). *Opisthioglyphe ranae, C. urniger* and *T. excavata* made the highest contributions to the dissimilarity. The dissimilarity between the infracommunities from lakes Telbin and Tiahle was comparatively moderate: ANOSIM *R* = 0.57 (*p* < 0.01); *O. ranae, P. confusus* and *C. urniger* made the highest contributions to the dissimilarity.

### Helminth component communities

Similarity between the 3 component communities was estimated using the Bray–Curtis index. The highest similarity was observed between helminth communities from Lake Tiahle and from Olzhyn Island (Bray–Curtis index 0.37). The similarity between helminth communities from Lake Telbin and Lake Tiahle was also relatively high (Bray–Curtis index 0.32). The lowest similarity was between helminth communities from Lake Telbin and Olzhyn Island (Bray–Curtis index 0.11). The results of the comparison appeared to be somewhat different from the comparison based only on the species composition (Sorensen similarity index) of the communities (see above), presumably due to the significantly different abundance of at least some of the common helminth species in the samples from Lake Telbin and Olzhyn Island: *I. neglecta, D. subclavatus, O. ranae* and *P. confusus* (see Table 2).

Species richness in helminth component communities, estimated using several methods (Table 4), appeared to be slightly higher than the actual number of species in the samples. *Chao1* and *bootstrap* estimators suggested the presence of 1 or 2 unrevealed species in each component community. According to the *jackknife* estimator, 4 more species might be present in the component communities from Lake Telbin and Lake Tiahle. In comparison, the component community from Olzhyn Island might include 2 more species, in addition to the 14 observed. All methods confirmed that the species richness in the helminth component community from Lake Tiahle was the highest. According to the *jackknife* estimator, the species richness in the component community from Lake Telbin is higher than that from Olzhyn Island (18 vs 16 species), despite the same number of species (14) being found in both samples. Moreover, Margalef’s index of species richness was the highest in the component community from Lake Telbin, apparently due to the much smaller total number of helminth specimens collected in this locality (Table 4).

**Table 4.** Indicators of species richness and diversity in helminth component communities of *Pelophylax ridibundus* from 3 localities Table 4 long description.

Indicator	Lake Telbin	Lake Tiahle	Olzhyn Island
Species richness:			
Observed	14	15	14
Estimated (*Chao1*)	14	16	14
Estimated (*jackknife1*)	18	19	16
Estimated (*bootstrap*)	15	17	15
Margalef’s index of species richness	1.80	1.78	1.52
Shannon diversity index	1.59	1.45	1.09
Gini–Simpson diversity index (1 − *λ*)	0.69	0.70	0.51
Pielou’s evenness index	0.60	0.54	0.41
Berger–Parker dominance index	0.51	0.41	0.66

Due to the lowest equitability (the highest dominance) in the structure of the helminth component community from Olzhyn Island, the diversity indices were the lowest there (Table 4). Shannon, Gini–Simpson and Pielou’s indices for the component communities from Lake Telbin and Lake Tiahle were similar. Interestingly, Pielou’s and Shannon indices for the community from Lake Telbin were slightly higher than those for the community from Lake Tiahle, despite the dominance identified by the Berger–Parker index being higher in the former community.

**Figure 5.**
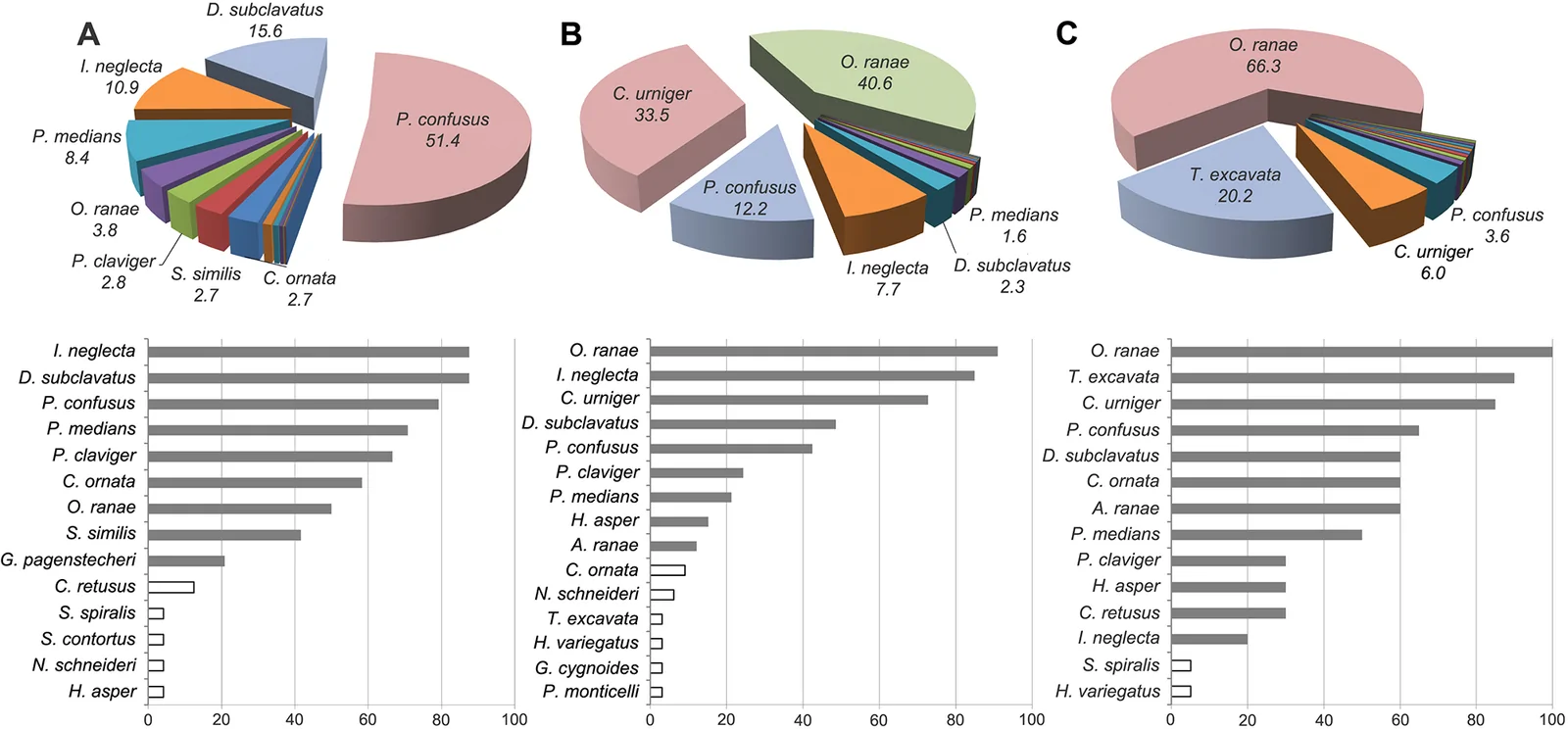
Relative abundance (pie charts, in %) and infection prevalence (bar charts, in %) of helminth species in samples of *Pelophylax ridibundus* from Lake Telbin (A), Lake Tiahle (B) and Olzhyn Island (C). Labelled on pie charts are only species with relative abundance higher than 1%. White bars indicate statistically uncertain species. Figure 5 long description.

In total, 14 helminth species were classified as statistically certain in at least one of the 3 localities based on the infection prevalence (minimum value of the confidence interval higher than the prevalence of infection corresponding to 1 infected host specimen in the sample): 12 in the sample from Olzhyn Island, 9 in the samples from Lake Telbin and Lake Tiahle (Table 2, Figure 5). Six statistically certain species were present in the samples from all 3 localities: the nematode *I. neglecta*, and the trematodes *D. subclavatus, O. ranae, P. medians, P. claviger* and *P. confusus*. No additional shared certain species were observed in the samples from Lake Telbin and Lake Tiahle, whereas the nematode *C. ornata* was among the certain species in both Lake Telbin and Olzhyn Island. Samples from Lake Tiahle and Olzhyn Island shared 9 certain species: the 6 mentioned above, plus the acanthocephalan *A. ranae*, and the trematodes *C. urniger* and *H. asper*.

In the sample from Lake Telbin, 4 species, namely *D. subclavatus, I. neglecta, P. medians* and *P. confusus*, occurred in more than 70% of frogs; their RAs were higher than 5% (Figure 5A). These 4 were assigned to the group of predominating species in the helminth community; their total RA was 86.3%. In the sample from Lake Tiahle, 3 species predominated based on their prevalence and RA (Figure 5B): *C. urniger, I. neglecta* and *O. ranae. Prosotocus confusus* was among the species with high RA (12.2%), while its infection prevalence was only 42.4%. Four species with the RA higher than 5% composed 94.1% of all helminth specimens in the sample. In the sample from Olzhyn Island, 3 species, namely *C. urniger, O. ranae* and *T. excavata*, predominated in the community due to the comparatively high infection prevalence (>80%) and the RA higher than 5% (Figure 5C). Specimens of these species composed a total of 92.5% of all helminths in the sample.

The composition of the groups of predominant helminth species in separate localities confirmed the highest differences between the component communities from Lake Telbin and Olzhyn Island: no species were shared by the predominant groups in these 2 localities. *Icosiella neglecta* and *P. confusus* were among the predominant species in Lake Telbin and Lake Tiahle. *Codonocephalus urniger* and *O. ranae* were among the predominant species in Lake Tiahle and Olzhyn Island. Therefore, none of the predominant species in the helminth community from Lake Tiahle was unique; all of them were found among the predominant species in the other 2 localities. *Tylodelphys excavata* was a predominant species only in the helminth community from Olzhyn Island; *D. subclavatus* predominated only in the helminth community from Lake Telbin. Interestingly, *O. ranae* was strongly predominant in the helminth communities from Lake Tiahle (infection prevalence 90.9%; RA 40.6%) and Olzhyn Island (infection prevalence 100%; RA 66.3%), while it was not among the predominant species in the helminth community from Lake Telbin (infection prevalence 50.0%; RA 3.8%).

## Discussion

In the present study, we found 20 helminth species in marsh frogs from 3 populations in Kyiv city. The number of species appeared to be the same as recorded in this host in our previous study, which was performed on a larger territory in northern Ukraine, including both urbanized and natural areas (Kuzmin et al., 2020). Such a number is notably smaller than that reported in a recent survey on the marsh frog from Vylkove (south of Ukraine) where 25 helminth species were recorded (Greben et al., 2025) and also smaller than those in similar studies from decades ago: Mazurmovich (1951) found 29 helminth species in the marsh frogs from Kyiv and Kaniv vicinities; Maguza (1972) recorded 26 helminth species in this host in northern Ukraine (Polissia [forest] region).

Species richness of helminths observed in separate populations of *P. ridibundus* in the present study (14–15 species) did not differ dramatically from the number of species recorded in similar studies, both in natural and urbanized areas. For instance, Rezvantseva et al. (2010) found 15 helminth species in the sample of marsh frogs in eastern Ukraine (Kharkiv region); Chihlaev et al. (2009) recorded from 15 to 23 helminth species in this host in 4 urbanized localities in the Volga basin, while Vershinin et al. (2017) recorded just 3–5 helminth species in *P. ridibundus* in urban and 10 species in suburban localities of Yekaterinburg agglomeration (Russia).

Urbanization may reduce the number of batrachophagous predators in a particular area and, consequently, decrease the diversity of larval helminths infecting amphibians. In the sample of helminths from Lake Telbin, we found only 1 such helminth species, namely *S. contortus*. For this nematode, cyclopid crustaceans are intermediate hosts, turtles are definitive hosts, while amphibians may serve as paratenic hosts (Sharpilo, 1976). We found larval stages of other helminth species in the 2 other localities: *P. monticelli* (parasite of grass snakes), *C. urniger* (parasite of bitterns) and *T. excavata* (parasite of storks) were present in the sample from Lake Tiahle; the latter 2 species were also found in the sample from Olzhyn Island. In natural habitats, the number of helminth species that use *P. ridibundus* as an intermediate or paratenic host may be much larger. Approximately half (14 out of 31) of helminth species recorded by Volgar-Pastukhova (1959) in marsh frogs from the vicinity of Vylkove (Odesa region, Danube Delta) were represented by larval stages. A more recent survey of helminths in the same locality (Greben et al., 2025) reported 8 species using *P. ridibundus* as an intermediate host.

In the present survey, *S. spiralis* is reported for the first time from amphibians in Ukraine. The species was described from green frogs (*Pelophylax* spp.) in Poland (Grabda-Kazubska, 1978). Presumably, the species has a scattered distribution in Europe. On the other hand, these nematodes may be easily overlooked during the examination of frogs, for they are small, transparent and dwell in the posterior intestine, embedded between the folds of intestinal epithelium.

*Strongyloides spiralis* is one of the 3 monoxenous parasites recorded in the present study. The other 2 are nematodes of the Cosmocercidae: *C. ornata* and *N. schneideri*. Of these 3 species, only *C. ornata* was among the statistically certain species in at least 2 localities, Lake Telbin and Olzhyn Island (see Figure 5). Other species showing comparatively high prevalence and abundance in separate communities and in the whole studied sample of helminths were all heteroxenous parasites. For instance, 6 statistically certain species recorded in all 3 localities were the nematode *I. neglecta* and the trematodes *D. subclavatus, O. ranae, P. medians, P. claviger* and *P. confusus*. They are all heteroxenous parasites with 1 (*I. neglecta, D. subclavatus* and *O. ranae*) or 2 (*P. medians, P. claviger* and *P. confusus*) intermediate hosts in their life cycles. Moreover, heteroxenous parasites were assigned to the groups of predominating species in all 3 localities: *D. subclavatus* in Lake Telbin, *I. neglecta* and *P. confusus* in Lake Telbin and Lake Tiahle, and *C. urniger* and *O. ranae* in Lake Tiahle and Olzhyn Island. These helminths apparently successfully tolerate habitat modifications caused by urbanization. This observation is an alternative to the previously suggested conclusion that in disturbed landscapes, parasites with direct life cycles are more common than those with complex life cycles (Marcogliese, 2023). We hypothesize, therefore, that environmental conditions allowing the survival of the *P. ridibundus* population are also conducive for the successful transmission of its most common heteroxenous parasites.

In Lake Telbin, where the degree of urbanization was the highest among the 3 studied localities, the species richness in the helminth community might be expected to be the lowest. Moreover, the malnutrition of hosts (see Figure 2) and morphological abnormalities in some helminth species (see Figure 3) were observed in this locality. However, the helminth species richness in the sample from Telbin was higher than that in the sample from Lake Tiahle (median 6 vs 4, see Table 3) at the infracommunity level, and higher than in the sample from Olzhyn Island, according to the *jackknife* estimator (18 vs 16, see Table 4), at the component community level.

Moreover, the diversity indices of the helminth component community from Lake Telbin were similar to, or even higher than, those of the helminth communities from the 2 other localities (Table 4). The helminth community from Lake Telbin differed from the 2 other ones mainly by the lowest helminth abundance, with just 57.5 helminth specimens infecting a host individual on average (see Table 3). Such low abundance was associated with higher evenness in the helminth community from Telbin, as evidenced by the highest Shannon and Pielou’s indices and the largest number of species with RA higher than 1% (8 in Telbin vs 6 in Tiahle and 4 in Olzhyn Island). We assume that while moderate habitat modification (= urbanization) decreases the evenness in helminth communities of *P. ridibundus* due to an increase in the abundance of some species, less susceptible to modifications (e.g. *O. ranae*), the more substantial modification may evenly decrease the abundance of all remaining species in the community and, consequently, increase the evenness. Further studies might verify the proposed scenario for helminth communities across other hosts and localities with varying degrees of urbanization.

## Figure 1 Long description

The map displays three collection sites in Kyiv, Ukraine, marked with red arrows and numbers. Site 1 is Lake Telbin, located on the eastern bank of the Dnipro River. Site 2 is Lake Tiahle, also situated on the eastern bank. Site 3 is Olzhyn Island, positioned further south along the river. The map includes a scale bar indicating 1 km for distance reference. An inset map shows the broader region of Kyiv Oblast, providing context for the location of these sites within the city and surrounding areas.

Navigate back to Figure 1.

## Figure 3 Long description

The image A showing a light micrograph labeled A of Cosmocerca ornata in a full-body view. A vertical scale bar is labeled 1 millimeter. The image B showing a light micrograph labeled B of the anterior part of the body in close-up. A vertical scale bar is labeled 0.2 millimeters. The image C showing a light micrograph labeled C of the posterior part of the body in close-up. The body is curved and a pointed end is shown. A vertical scale bar is labeled 0.4 millimeters. The image D showing a light micrograph labeled D of the cuticle surface in close-up, showing a dense textured area and a narrow vertical band near the center. A horizontal scale bar is labeled 0.02 millimeters.

Navigate back to Figure 3.

## Figure 4 Long description

Insufficient visual information to describe this element accurately.

Navigate back to Figure 4.

## Figure 5 Long description

The image contains three sets of pie and bar charts labeled A, B and C. Each set represents helminth species data from different locations. Set A: Lake Telbin - Pie chart shows relative abundance in percent: P. confusus 57.4, D. subclavatus 15.6, I. neglecta 10.0, P. medians 8.4, P. claviger 3.8, S. solidus 2.7, C. ornata 2.7. - Bar chart shows infection prevalence in percent: D. subclavatus 80, P. confusus 70, P. medians 60, P. claviger 50, C. ornata 40, O. ranae 30, S. solidus 20. - Dominant species in abundance are also prevalent. Set B: Lake Tiahle - Pie chart shows relative abundance in percent: O. ranae 49.6, C. urniger 33.5, P. confusus 12.1, I. neglecta 7.7, P. medians 3.2, D. subclavatus 3.2. - Bar chart shows infection prevalence in percent: O. ranae 90, I. neglecta 80, C. urniger 70, D. subclavatus 60, P. confusus 50. - O. ranae dominates both abundance and prevalence. Set C: Olzhyn Island - Pie chart shows relative abundance in percent: O. ranae 63.3, T. excavatum 20.2, P. confusus 6.0, C. urniger 6.0. - Bar chart shows infection prevalence in percent: O. ranae 95, T. excavatum 85, C. urniger 75, P. confusus 65. - O. ranae is most abundant and prevalent. The pie charts illustrate species composition, while the bar charts show infection prevalence. Dominant species in abundance often match those with high prevalence. Consistent color mapping is used across panels to differentiate species visually.

Navigate back to Figure 5.

## Table 1 Long description

The table summarizes marsh frog sampling at three localities, listing coordinates, collection months and years, numbers of females and males, and host snout-vent length in millimeters. Lake Telbin was sampled in September of 2018 and 2019 with 24 frogs, 8 females and 16 males, and an average length of 82 mm with a range from 58 to 102. Lake Tiahle was sampled from June through September 2020 with 33 frogs, 16 females and 17 males, averaging 74 mm and ranging from 43 to 99. Olzhyn Island was sampled in July 2021 with 20 frogs, evenly split 10 females and 10 males, averaging 68 mm and ranging from 47 to 92. Overall, Lake Telbin frogs were largest on average, while Olzhyn Island frogs were smallest on average. The sites differ in sampling periods and years, so size differences may reflect season, year, or site conditions rather than a single cause.

Navigate back to Table 1.

## Table 2 Long description

The table reports infection prevalence (percent infected), mean abundance, and infection intensity for 20 helminth taxa in marsh frogs from Lake Telbin (24 frogs), Lake Tiahle (33), and Olzhyn Island (20), with confidence intervals for prevalence and mean abundance and ranges and medians for intensity. Olzhyn Island stands out for extreme trematode infection by Opisthioglyphe ranae: prevalence is 100 percent and mean abundance is about 173 parasites per frog, far higher than Lake Tiahle (about 33) and Lake Telbin (about 2). Metacercariae of Codonocephalus urniger are also common and heavy at Lake Tiahle and Olzhyn Island, reaching roughly 73 percent prevalence with mean abundance about 27 at Lake Tiahle and 85 percent with mean abundance about 16 at Olzhyn Island, while absent at Lake Telbin. Tylodelphys excavata metacercariae are absent at Lake Telbin, rare at Lake Tiahle, but very common at Olzhyn Island (about 90 percent prevalence) with high mean abundance (about 53). Several adult trematodes are most prominent at Lake Telbin, including Prosotocus confusus (about 79 percent prevalence; mean abundance about 30) and Diplodiscus subclavatus (about 88 percent; mean abundance about 9), both lower at the other sites. Among nematodes, Icosiella neglecta is very frequent at both lakes (about 85 to 88 percent prevalence; mean abundance about 6) but much less common at Olzhyn Island (about 20 percent). Acanthocephalus ranae occurs only at Lake Tiahle and Olzhyn Island, with higher prevalence at Olzhyn Island (about 60 percent) than Lake Tiahle (about 12 percent). Comparisons should consider the different sample sizes and the uncertainty shown by confidence intervals, and intensity values vary widely within sites as indicated by ranges and medians.

Navigate back to Table 2.

## Table 3 Long description

The table summarizes helminth infracommunity species richness and total abundance in marsh frogs at three localities, reported as mean, range, and median. Species richness averages 5.9 at Lake Telbin (range 2 to 8; median 6), 4.4 at Lake Tiahle (range 1 to 9; median 4), and 6.9 at Olzhyn Island (range 3 to 11; median 7). Abundance averages 57.5 at Lake Telbin (range 2 to 129; median 51.5) and 80.2 at Lake Tiahle (range 1 to 246; median 72). Olzhyn Island has much higher mean abundance at 260.4, with a very wide range from 17 to 2,871 and a median of 98. The large gap between mean and median at Olzhyn Island suggests a few frogs with extremely high parasite counts, so medians may better reflect typical abundance there.

Navigate back to Table 3.

## Table 4 Long description

The table compares helminth community species richness and diversity indicators for Pelophylax ridibundus across Lake Telbin, Lake Tiahle, and Olzhyn Island. Observed species richness is similar across sites: 14 at Lake Telbin, 15 at Lake Tiahle, and 14 at Olzhyn Island. Estimated richness varies more, with the highest estimates at Lake Tiahle (Chao1 16, jackknife1 19, bootstrap 17) and the lowest at Olzhyn Island (Chao1 14, jackknife1 16, bootstrap 15). Margalef’s richness index is highest at Lake Telbin (1.80), close at Lake Tiahle (1.78), and lower at Olzhyn Island (1.52). Diversity is highest at Lake Telbin (Shannon 1.59; Gini-Simpson 0.69) and similar at Lake Tiahle (Shannon 1.45; Gini-Simpson 0.70), while Olzhyn Island is lower (Shannon 1.09; Gini-Simpson 0.51). Evenness follows the same pattern, with Pielou’s index 0.60 at Lake Telbin, 0.54 at Lake Tiahle, and 0.41 at Olzhyn Island. Dominance is greatest at Olzhyn Island (Berger-Parker 0.66), indicating one species is more prevalent there than at Lake Telbin (0.51) or Lake Tiahle (0.41). Because richness estimates depend on sampling and estimator choice, comparisons should consider that observed counts are similar while diversity and dominance differ more strongly.

Navigate back to Table 4.
